# Expanded Quality Management Using Information Power (EQUIP): protocol for a quasi-experimental study to improve maternal and newborn health in Tanzania and Uganda

**DOI:** 10.1186/1748-5908-9-41

**Published:** 2014-04-02

**Authors:** Claudia Hanson, Peter Waiswa, Tanya Marchant, Michael Marx, Fatuma Manzi, Godfrey Mbaruku, Alex Rowe, Göran Tomson, Joanna Schellenberg, Stefan Peterson

**Affiliations:** 1 Department of Public Health Sciences, Karolinska Institutet, Stockholm, Sweden; 2 Department of Disease Control, London School of Hygiene and Tropical Medicine, London, United Kingdom; 3 Makerere University, College of Health Sciences, School of Public Health, Kampala, Uganda; 4 Evaplan GmbH the University of Heidelberg, Heidelberg, Germany; 5 Ifakara Health Institute, Dar-es-Salaam, Tanzania; 6 Malaria Branch, Division of Parasitic Diseases and Malaria, Center for Global Health, Centers for Disease Control and Prevention, Atlanta, USA; 7 Department of Learning, Informatics, Management, Ethics; Karolinska Institutet, Stockholm, Sweden; 8 Department of Women’s and Children’s Health, International Maternal and Child Health Unit, Uppsala University, Uppsala, Sweden

**Keywords:** Quality management, Quality improvement, Maternal and child health, Health system strengthening, Community empowerment, Tanzania, Uganda

## Abstract

**Background:**

Maternal and newborn mortality remain unacceptably high in sub-Saharan Africa. Tanzania and Uganda are committed to reduce maternal and newborn mortality, but progress has been limited and many essential interventions are unavailable in primary and referral facilities. Quality management has the potential to overcome low implementation levels by assisting teams of health workers and others finding local solutions to problems in delivering quality care and the underutilization of health services by the community. Existing evidence of the effect of quality management on health worker performance in these contexts has important limitations, and the feasibility of expanding quality management to the community level is unknown. We aim to assess quality management at the district, facility, and community levels, supported by information from high-quality, continuous surveys, and report effects of the quality management intervention on the utilization and quality of services in Tanzania and Uganda.

**Methods:**

In Uganda and Tanzania, the Expanded Quality Management Using Information Power (EQUIP) intervention is implemented in one intervention district and evaluated using a plausibility design with one non-randomly selected comparison district. The quality management approach is based on the collaborative model for improvement, in which groups of quality improvement teams test new implementation strategies (change ideas) and periodically meet to share results and identify the best strategies. The teams use locally-generated community and health facility data to monitor improvements. In addition, data from continuous health facility and household surveys are used to guide prioritization and decision making by quality improvement teams as well as for evaluation of the intervention. These data include input, process, output, coverage, implementation practice, and client satisfaction indicators in both intervention and comparison districts. Thus, intervention districts receive quality management and continuous surveys, and comparison districts-only continuous surveys.

**Discussion:**

EQUIP is a district-scale, proof-of-concept study that evaluates a quality management approach for maternal and newborn health including communities, health facilities, and district health managers, supported by high-quality data from independent continuous household and health facility surveys. The study will generate robust evidence about the effectiveness of quality management and will inform future nationwide implementation approaches for health system strengthening in low-resource settings.

**Trial registration:**

PACTR201311000681314

## Background

Maternal and newborn mortality remain unacceptably high in sub-Saharan Africa, with levels of 500 per 100,000 live births and 30 per 1,000 live births, respectively [1,2]. Global progress in reducing maternal and newborn mortality is at 3.1% and 2.1% per year, respectively, far below the MDG targets of 5.5% and 4.5% [2,3]. Globally 287,000 maternal deaths, between 3.1 and 3.6 million newborn deaths and 2.6 million stillbirths occur each year [2,4,5], many of which could be prevented by technically simple and affordable interventions as proposed by the World Health Organisation (WHO) [6]. Most of these interventions are taught and promoted globally as part of medical pre-service and in-service training [7], but are still not implemented at scale [8]—a situation sometimes referred to as the know-do gap [9]. In Tanzania and Uganda, many essential interventions such as active management of third stage of labor are not implemented at scale, and availability of essential items for infection prevention are missing in facilities [10,11].

Quality management (QM) strategies are increasingly promoted to close this know-do gap, and simultaneously to strengthen health systems [12]. QM involves applying a set of principles to improve quality: conceptualizing work as processes (*e.g.*, following a case-management guideline), designing processes to reduce errors, focusing improvement efforts on the most vital processes, satisfying both clients and employees, monitoring quality, using scientific and statistical thinking, creating new organizational structures (*e.g.*, quality improvement teams), and involving all workers in quality improvement. QM also includes a structured problem-solving methodology, which uses teams to improve quality with continuous plan-do-study-act (PDSA) cycles, which monitor indicators, identify problems, understand causes, implement solutions, check if solutions are working, and modify solutions as needed. This problem-solving approach (called the quality improvement process, among other names) was first used on a large scale in the automobile manufacturing industry in Japan [13] and has increasingly entered the medical field since the 1980s [14-16], being used within primary healthcare and in low- and middle-income countries [17].

The improvement collaborative approach, which has been used in multiple low- and middle-income countries [18], brings together groups of health professionals from multiple health facilities to work in a structured way to improve one aspect of the quality of their service [19].

Using a feedback loop of relevant data is an essential aspect of any QM strategy [20], but little is known about how the feedback loop should best be operationalized [21,22], particularly in low-income settings. One problem is that high-quality, timely data are frequently not available at the local level. Data from Demographic and Health Surveys (DHSs) or Multiple Cluster Indicator Surveys (MICSs) are considered a gold standard for information on coverage and health outcomes but typically cannot report at the district level, and are only generated at three- to five-year intervals. Thus their relevance for QM is limited.

Research groups have reported on the potential of continuous surveys to generate high-quality level data for the district level. The continuous survey approach applies repeated sampling at regular intervals, enabling analysis and reporting of survey data at the district level, timed to support implementation processes as well as endline assessments [23,24]. Given their ability to provide district-level data at more frequent time intervals, continuous surveys might support QM processes.

QM in health has typically not engaged healthcare consumers actively. While not strictly a QM intervention, but more one of social accountability, a randomized controlled trial in Uganda showed how the feedback of high-quality local health information can be used to empower communities to advocate for change. Data collected through a household survey were made available to communities in facilitated sessions using citizen report cards including information on the quality of care provided at the nearest health facility. The intervention showed an impact both on the quality of health services and uptake of care [25], and thus gave evidence that making data on quality and utilization of health services available to communities could lead to improvements.

Despite recognition of the potential of QM to improve health outcomes, only few high-quality studies from low-income countries are available. The most recent Cochrane review on audit and feedback reported positive effects on professional behavior and outcomes, but included only very few studies from low-income countries [20]. There is some compelling evidence suggesting that improvement collaborative might be effective in improving implementation of essential interventions, but there are few high-quality studies [18]. Rarely has QM been assessed using independent data and across the breadth of its components; additionally, high-quality evidence from low-income settings is particularly scarce. Also, to the best of our knowledge, no rigorous evaluation of a QM strategy is available based on independently generated data from a low-income setting where structural deficiencies in terms of drugs, supplies, and staffing—which are largely out of control of local teams—are prominent barriers to the provision of high-quality care. Finally, to the best of our knowledge, no evidence has been reported of an integrated QM approach that includes management, supply-side and demand-side quality improvement teams (QITs).

The EQUIP hypothesis is that a QM approach expanded to district, facility and community level and supported by report cards generated through continuous household and health facility surveys could have a measurable impact on maternal and newborn health in high-mortality settings of Tanzania and Uganda. The specific objectives are:

1. To assess the effects of the EQUIP intervention on uptake and quality of care of key maternal and newborn health interventions;

2. To assess the feasibility and acceptability of the intervention;

3. To model the potential impact of the intervention using the Lives Saved Tool (LiST);

4. To estimate cost and cost-effectiveness of the intervention.

## Method

### Study design

EQUIP is designed as a plausibility study where we compare district-level estimates in intervention and comparison districts with respect to change in utilization and quality of healthcare [26]. During the entire study period, ongoing data collection via continuous, high-quality household and heath facility surveys is used to estimate pre- and post-intervention outcome levels in one intervention and one non-randomly selected comparison district each in Uganda and Tanzania. The continuous household surveys and health facility censuses cover implementation and comparison districts. The QM intervention, supported by report cards using data generated by the continuous surveys, is implemented in intervention districts only. For evaluation, changes over time in quality and uptake of key maternal and newborn interventions in intervention areas are compared with changes over time in comparison areas, with careful attention paid to contextual factors that also vary over time [27]. Such contextual factors may be potential confounders of the relationship between the EQUIP intervention and study outcomes. The district was chosen as a unit of implementation because in both countries, planning and implementation has been decentralized to the district level, and EQUIP is designed to be implemented through the district structure.

### The study sites

EQUIP is implemented in eastern Uganda (Mayuge District) and southern Tanzania (Tandahimba District); two neighbouring districts serve as comparison areas (Namayingo District in Uganda and Newala District in Tanzania). All four districts have high maternal and newborn mortality rates and are predominantly rural with small district capitals (see Table 1). Mayuge District has a population of about 400,000, lies along the northern shores of Lake Victoria and includes six islands, however, the islands are not included in the intervention due to resource limitations. Namayingo District has a similar geography but only half of the population that Mayuge has. Tandahimba and Newala Districts are both situated on the Makonde plateau in Mtwara Region of the southern zone of Tanzania, both with a population slightly over 200,000 people.

**Table 1:** Main health indicators in the intervention and comparison areas

**Indicator**	**Mayuge (intervention)**	**Namayingo (comparison)**	**Tandahimba (intervention)**	**Newala (comparison)**
Population	412,500^1^	233,000^1^	227,514^2^	205,492^2^
Administrative structure	3 health sub-districts, 13 sub-countries 521 (488) villages*	2 health sub-districts, 270 villages	3 divisions, 30 wards 157 villages	5 divisions, 29 wards, 155 villages
Health facilities ~	41 + 1 hospital	22 (no hospital)	33 + 1 hospital	30 + 1 hospital
Maternal mortality ratio	438 (national, 7 years prior to survey)^3^		712 (95% CI 652-777) (2004–2007)^5^	
Newborn mortality rate	23 (2001–2011, East Central)^3^		31 (2001–2010, Southern Zone)^4^	
Infant mortality rate	61 (2001–2011, East Central)^3^		68 (2001–2010, Southern Zone)^4^	
< 5 mortality rate	106 (2001–2011, East Central)^3^		94 (2001–2010, Southern Zone)^4^	
Total fertility rate	6.8 (rural Uganda)^3^		4.4 (2010, Southern Zone)^4^	
HIV prevalence	5.9% (East Central)^6^		4.1% (2011–12, Mtwara region)^6^	
Institutional Delivery	67% (2011, East Central)^3^		59% (2010, Mtwara region)^4^	
Antenatal care attendance 1+	91% (2011, East Central)^3^		99% (2010, Mtwara region)^4^	
Antenatal Care attendance 4+	46% (20011, rural Uganda)^3^		43% (2010, Tanzania)^4^	

*The intervention is implemented in 488 villages. 25 villages on islands were excluded because resources available for the project did not allow inclusion of these difficult to reach population, thus also three health facilities were excluded.

~The status of the health facilities describes the situation in October 2013. Health facilities that did not offer reproductive and child health services were not included in the quality management intervention or in the survey, and new facilities were successively added (38, 22, 32 and 30 facilities were included in the continuous survey in Mayuge, Namayingo, Tandahimba and Newala in the first three rounds and the quality improvement work was ongoing in 30 facilities in Mayuge and 32 facilities in Tandahimba in October 2013).

^1^Uganda Bureau of Statistics, 2009 mid-year projection.

^2^Census 2012, The United Republic of Tanzania: 2012 Population and Housing Census. Population Distribution by administrative areas. In.: National Bureau of Statistics, Dar-es-Salaam. Office of Chief Government Statistician, Zanzibar; March, 2013.

^3^Uganda DHS 2012 Uganda Bureau of Statistics (UBOS), ICF International Inc: Uganda Dempgraphic and Health Survey 2011. In. Edited by UBOS and ICF Inc. Kampala, Uganda and Calverton, Maryland, 2012.

^4^Tanzania DHS 2010. National Bureau of Statistics (NBS) Tanzania, ICF Macro: Tanzania Demographic and Health Survey. In. Dar-es-Salaam, Tanzania: NBS and ICF Macro,; 2011.

^5^Census in five districts in southern Tanzania, Hanson C: The epidemiology of maternal mortality in southern Tanzania. London, UK, http://researchonline.lshtm.ac.uk/1012993/: London School of Hygiene and Tropical Medicine; April 2013.

^6^AIDS Indicator survey 2011 in Uganda, Ministry of Health, ICF International, Centers for Disease Control and Prevention, U.S. Agency for International Development, WHO Uganda Maryland 2012 and Tanzania HIV/AIDS and Malaria Indicator Survey 2011-2012 .Tanzania Commission for AIDS (TACAIDS); Zanzibar AIDS Commission (ZAC); National Bureau of Statistics (NBS); Office of the Chief Government Statistician (OCGS), ICF International 2013: Tanzania HIV/AIDS and Malaria Indicator Survey 2011-12. In. Edited by National Bureau of Statistics (NBS) Tanzania. Dar es Salaam, Tanzania: TACAIDS, ZAC,NBS, OCGS, and ICF International,; 2013.

In both study areas, most people are subsistence farmers. Mud-walled houses with thatched roofs are still common. The road network consists of a few tarmac or gravel roads and smaller roads and paths. Many places are difficult to reach by car because of poor road conditions, particularly in the rainy season.

### The EQUIP intervention

**Figure 1:**
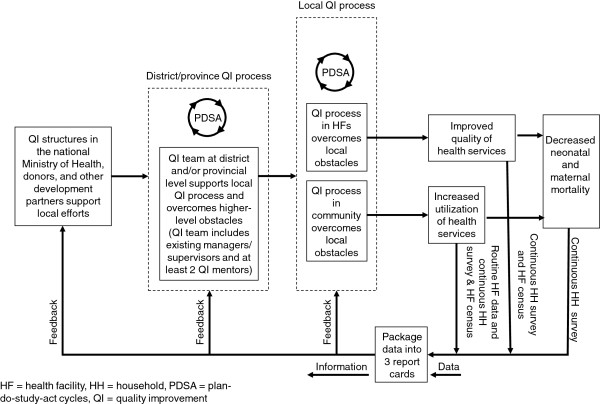
The EQUIP quality management approach.

The conceptual framework for the EQUIP intervention, which combines QM and report cards generated through continuous surveys is shown in Figure 1.

EQUIP’s QM intervention is based on the quality improvement process (or ‘model for improvement’), which is a short-term rapid learning approach to seek improvement in a focused area [19,28]. This approach incorporates three defining questions and the PDSA cycle. The questions are: What are we trying to accomplish? How will we know that change is an improvement? What changes can we make that will result in an improvement? PDSA cycles guide QITs in identifying problems, defining a strategy that can produce change (a change idea), and testing the strategy using locally generated data to determine if the change is an improvement [28]. The driving vision behind this approach is that sound evidence exists of what needs to be done to improve outcomes and reduce costs, but that it is not used in daily work.

**Figure 2:**
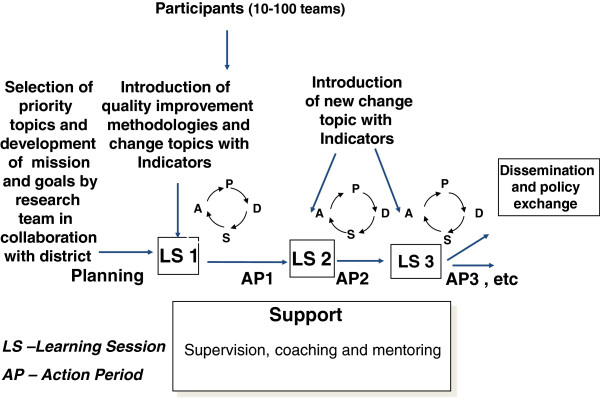
**Model of improvement (adapted from Institute for Healthcare Improvement.** The Breakthrough Series, 2003 [19]).

To implement the quality improvement process in a way that can more rapidly find solutions to difficult obstacles and scale-up improvement in multiple health facilities (or other sites), EQUIP uses the collaborative model for improvement (Figure 2) [19,29]. This model brings together QITs from multiple sites to work on the same obstacle. Action periods, lasting several months—when QITs are using PDSA cycles in their sites to test change ideas—alternate with learning sessions—one-day workshops when QITs meet to compare results and work towards developing a change idea that can be used across all sites. The EQUIP study team provides coaching and mentoring during action periods and facilitates the learning sessions (find details in Annex II).

A key innovation of EQUIP is that QM is expanded to three levels: district health managers; health facility staff; and members of the communities accessing services (Figure 1). QITs are formed at all these levels and mentored to use PDSA cycles to overcome local barriers. District-level teams are mentored to work on strategic decisions and human resource planning and to overcome supply barriers (*e.g.*, medicine stock-outs). Facility QITs are guided to overcome local barriers to delivering key maternal and newborn interventions, and to increase demand by improving client satisfaction (*e.g.*, by treating clients well). Community QITs are guided to primarily focus on increasing utilization of services at facilities (creating demand) and to improve knowledge of community members in regard to specific maternal and newborn healthcare practises.

QITs monitor progress through local data collection as part of their ongoing PDSA cycles. Data collected include process indicators that reflect the desired improvements, for example, at health facilities, whether blood pressure is measured during antenatal care visits, or whether women delivered in a health facility. EQUIP mentors train QITs to generate run charts that display data on process indicators over time (usually by month) (see example in Annex III).

The report card innovation synthesises district-level data generated by the continuous surveys as reported by Marchant et al. The use of continuous surveys to generate and report high quality timely maternal and newborn health data at the district level in Tanzania and Uganda, submitted.

The household survey applies repeat probability sampling to select ten household clusters per district each month for a period of 30 months. In addition, once every four months, a census of all health facilities in the study districts is completed. The surveys measure input, process, output, coverage, implementation practice, and client satisfaction indicators [30] along the continuum of care from pre-pregnancy to the end of the post-partum period. Survey questionnaires are based on well-established sequences of questions as used in DHSs, MICSs, and service provision assessments (SPAs) and are based on earlier work in southern Tanzania [31].

Using these data, report cards are generated every four months in intervention districts addressing topics relevant to QITs. Report cards present several graphs printed on double-sided A4-size paper. Report cards are different for the district, health facility, and community levels—each tailored to relevant improvement topics and amount of detail that would be expected to be understood. The use of the report cards is facilitated by the EQUIP coordinators and district mentors. The data from report cards also facilitates the policy dialogue with the Ministry of Health [32].

### Implementation strategy

The implementation strategy in intervention districts in Uganda and Tanzania is aligned within government health structures. The main implementers are the district health management teams. Additionally, in Tanzania, the Department for community is included in the strategy to support the community level EQUIP activities (see Annex II).

In both countries, the EQUIP research project employs one medical doctor and one social scientist in each site. These two EQUIP coordinators organize, lead and assess the QM work (see Figure 1 and 2 in Annex II). The EQUIP coordinators have been supported during the set-up phase of the project by external QM coordinators. These two EQUIP coordinators train other mentors who are part of the government district health teams, guiding the prioritisation of the improvement work topics, preparing and facilitating the learning sessions, and giving continuous support to the QITs in health facilities and communities.

The EQUIP approach was developed and piloted during a one-year preparation phase (Nov 2010 to Oct 2011). This period included a four-day QM training for EQUIP project members and selected district staff led by international QM experts. Training of other staff, including those involved in mentoring and coaching, was provided during introductory workshops and learning sessions.

In both countries, at the district level, the QIT is comprised of the entire district health management team. Facility QITs include all health workers (range two-to-twelve staff). At the community level, teams include two volunteers selected from each village who form a team with neighboring villages, aggregated to a sub-district level to make improvement collaboratives with 80 and 100 members in Uganda and Tanzania, respectively.

QITs are trained in QM methods and use the PDSA cycles to structure their improvement work by: identifying problems and their underlying causes, identifying possible solutions, and implementing solutions and monitoring whether changes result in better outcomes at the level of individual QIT implementation.

The implementation period included four months—from July to Oct 2011—of testing the whole QM approach in a single sub-district. Immediately following this, QIT were formed and trained in all health facilities and all villages in the intervention districts over an eight- to twelve-month period.

During the learning sessions and supervision visits, the teams are encouraged to use various methods such as brainstorming, fishbone analysis, and other mapping techniques to identify the root causes of the problems. Based on these problems, local solutions to overcome barriers towards maternal and newborn healthcare provision and care-seeking are identified [28]. The main improvement work is included in daily activities in health facilities, also called the action period (see definition in Annex IV). Teams implement strategies for QM, also called change ideas, as a day-to-day activity and monitor their work with agreed indicators reflecting the change topic. QITs at the district, health facility, and community levels are advised to meet at least monthly, or preferably every two weeks. During these meetings, the teams analyze progress by reviewing their run-charts and as necessary, they adapt change ideas to overcome low implementation levels accordingly.

Teams are supported during the action phase by monthly coaching and mentoring visits that are conducted by district mentors together with EQUIP project members. Teams are also supported during quarterly one-day learning sessions held at the sub-district level. Learning sessions introduce a new topic related to maternal and newborn health and facilitate group discussions with the aim of developing improvement strategies to these new topics. Peer-to-peer exchange on improvement strategies and competition among QIT are encouraged in these sessions, thus leveraging the power of collective learning.

### Quality improvement topics

The selection of improvement topics introduced in learning sessions is guided by evidence-based recommendations from WHO and partners on essential interventions, commodities and guidelines [6,7] as well as country policy papers [33-35]. In a consultative process, priority ranking lists are prepared for topics amenable to implementation at five different levels of the healthcare system: community; lower level first-line facilities (health center II in Uganda and dispensaries in Tanzania); higher level first-line facilities (health center III and IV in Uganda, health centers in Tanzania); hospitals; and the district management structure.

### Selection of study sites

The intervention and comparison districts were purposively selected from districts where EQUIP researchers had established working relationships with key stakeholders to ensure that key district staff are likely to support the QM approach. Selection criteria for the intervention and comparison district were that: the districts are typical rural districts with limited human and financial resources; the intervention and comparison districts should be in the same region; the districts should be of comparable size with similar health infrastructure (such as the availability of a district hospital) and no other major QM activities going on; and no major differences in outcome indicators in the intervention and comparison districts are seen (see Table 1). However, in Uganda, the selected comparison district was split into two shortly after the selection process, leaving the comparison district with a smaller sample size and without a district hospital.

Uganda and Tanzania both have a pyramidal district health system. The population is served by a network of public facilities, with few private-not-for-profit (mission) facilities that are supported by the district health system. Mayuge District in Uganda has two private health facilities. The district health structure in both intervention districts is comprised of a hospital (in Mayuge a private-not-for-profit hospital and in Tandahimba a public hospital), a few higher-level health centers, and about 30 lower level facilities (34 in Mayuge District and 30 in Tandahimba District).

Both countries include some health structure at community level such as health agents or village health teams, but functionality varies. In both Tanzania and Uganda, health planning and implementation is decentralized as part of the local government reform in the past 10 years. In Tanzania funding is made available directly to districts through ‘basket funding’ which gives the district health management committees some level of autonomy to engage local priorities [11].

### Evaluation methodology

The evaluation compares intervention and comparison districts with respect to change in utilization and quality of healthcare using indicators of coverage, service quality and knowledge. It is based on a continuous survey of a total of 18,000 households and six repeat health facility censuses implemented between November 2011 and April 2014 in both intervention and comparison districts, including birth histories with women in reproductive age. Details are given by Marchant et al. The use of continuous surveys to generate and report high quality timely maternal and newborn health data at the district level in Tanzania and Uganda. Submitted.

Briefly, questionnaires are adapted from tools including the Safe Motherhood Needs Assessment, DHSs, MICSs, SPAs, and others [36-38]. A modular checklist-type questionnaire is used to assess health facilities, including staff employed, drugs, supplies and equipment and implementation of essential interventions for routine childbirth care using a ‘last event’ approach where health workers are asked to report on the care they provided during the last birth they attended. The household questionnaire has a module that includes questions on household assets, housing type, ethnic group and geographical position. Women of reproductive age (13 to 49 years in Tanzania and 15 to 49 in Uganda) are asked about knowledge and use of family planning and a pregnancy history since January 2010. Also, information on perceived quality of care is collected. Women with a live birth in the two years before the survey are asked about care received during the antenatal period, delivery and the post-partum period.

Regular documentation of contextual factors is conducted to support the plausibility evaluation (see Annex V). Changes in availability of financial and human resources, introduction of new policies or changes in procurement, other project and program activities in health and any major event or disruption of services are documented on a quarterly basis in both intervention and comparison districts [27,39].

A qualitative sub-study on feasibility and acceptability includes: how, when, and with what intensity the intervention is implemented in the intervention district; how the intervention worked at different levels; and changes and observations reported by QITs. In-depth interviews with district staff involved in the project are used to assess the acceptability of the QM approach and feasibility of implementation within the district structure.

The evaluation uses a non-interrupted time-series approach [40] to compare changes over time in primary outcomes (see below) in intervention and comparison areas. We generate a single estimate of effect for each primary outcome, adjusting for confounding factors and baseline levels. Provided that utilisation, quality or coverage improves sufficiently for an effect on survival to be plausible, we will also use the Lives Saved Tool (LiST) to model the potential impact of the intervention on child survival [41].

The primary coverage outcomes are

% of women delivering in a health facility (institutional delivery)

% of livebirths breastfed within one hour after delivery (immediate breastfeeding)

The primary **quality outcome** is

% of livebirths breastfed within one hour after delivery (immediate breastfeeding)

The primary **knowledge outcome** is

% of women knowing danger signs for pregnancy and newborn babies

Secondary outcomes on coverage, quality, and knowledge across the continuous of care are given by Marchant et al. The use of continuous surveys to generate and report high quality timely maternal and newborn health data at the district level in Tanzania and Uganda, submitted.

### Sample size

The sample size generated combining two rounds of data collection is sufficient to detect a 15% change in the two primary indicators: delivery in a health facility and breastfeeding within an hour, with 80% power and adjusted for a design effect (1.4) and refusals (10%). A 20% change could be detected with 80% power using one round of data collection for the two primary indicators (design effect of 1.4 and 10% refusals). See Marchant et al. The use of continuous surveys to generate and report high quality timely maternal and newborn health data at the district level in Tanzania and Uganda, submitted for a more detailed discussion.

### Economic evaluation

The economic evaluation uses a societal perspective to estimate cost-effectiveness of the intervention [42,43]. Data are collected as part of the continuous surveys and complemented by observational studies. Costs are classified by resource inputs (recurrent and capital) and by project activities. Economic costs reflect the full local value of resources used to implement the intervention (opportunity costs). The approach taken also involves the valuation of any non-remunerated time inputs such as additional health worker time.

Incremental cost-effectiveness ratios is evaluated in terms of costs per maternal and newborn deaths averted (as estimated using the LiST-tool) and the cost per health outcome such as cost per additional mother receiving quality skilled attendance. Scenario analysis is conducted to estimate the costs of replicating and scaling-up of the intervention.

### Ethical clearances

Ethical clearance for the study was obtained from the local and institutional review boards from Ifakara Health Institute through the Commission for Science and Technology, (NIMR/HQ/r.8a/Vol.IX/1034), the Makerere University School of Public Health and Uganda National Council of Science and Technology, and the London School of Hygiene and Tropical Medicine (LSHTM), ethical clearance No 5888. Extensive meetings were held at the start of the project to inform the district and sub-district authorities about the project and to obtain their consent for implementation. For the household survey, written consent to participate is obtained from the household head and from women of reproductive age.

### Trial status

The trial is still ongoing.

## Discussion

EQUIP aims to test whether a QM approach at three levels of care and supported by district level report cards generated by continuous surveys can improve the quality and utilization of services for mothers and newborns.

### Innovation and potential impact

EQUIP is designed to be a district-scale proof of concept study to evaluate the potential of QM, supported by data generated from continuous health facility and household surveys, to overcome barriers in health service delivery in two under-resourced district health systems in rural Africa. The project evaluates the effect of an information-driven QM process aiming for locally initiated quality improvements and capacity building with increased accountability towards communities and clients. This systemic QM model, which extends to communities, health facilities and districts, could be a model for health system strengthening in low-resource settings. The intervention responds to the World Health Report 2008 call to develop innovative ways of capacity building for change.

EQUIP is the first application of continuous surveys with continuous reporting for QM across four districts in sub-Saharan Africa, offering the opportunity to use data both to guide the intervention and for evaluation of effects. This approach offers an alternative paradigm to stand-alone cross-sectional surveys done every three to five years, which are primarily designed to inform international and national stakeholders [24]. Surveys that can produce reasonably precise indicator estimates at sub-national levels could have much more relevance for day-to-day operations and district or regional planning.

### Methodological considerations

As with the evaluation of other complex health interventions implemented at the district level, a randomised design is not feasible. Further, the study design compares intervention districts with QM and continuous surveys with facilitative feedback of survey results in the form of report cards every four months, versus comparison districts with continuous surveys only summarized in a written report sent to district health managers every 12 months. Our assumption is that the latter has no appreciable effect. Contextual factors, such as a change in district health services or other ongoing projects and programs, may modify or confound observed intervention effects and their careful assessment plays an important role in the EQUIP evaluation [27]. In addition, the feasibility and acceptability study will provide knowledge on how the intervention worked and which changes it has stimulated within the district health system. The dual use of data for both intervention and evaluation presupposes strong methods and the independence of the survey teams from the implementers [24]. The field interviewers are trained to not consider the intervention versus comparison status when collecting data, but the survey team is not blinded to the intervention, which presents a limitation. Finally, the continuous household surveys are not designed to measure mortality outcomes, but these will be estimated indirectly using the LiST model [44,45]. It will likely not be possible to separate the effects of sub-components such as the community or health facility component.

In conclusion, EQUIP is a district level proof-of-concept study that will evaluate a QM approach for maternal and newborn health including communities, health facilities and district health managers, supported by high-quality data from independent household and health facility surveys. The study will generate robust evidence about the effectiveness of QM and will inform future nationwide implementation approaches for health system strengthening in low-resource settings.

## Abbreviations

EQUIP: Expanded Quality Management Using Information Power; QM: Quality management; PDSA: Plan-Do-Study-Act; QIT: Quality improvement teams; DHS: Demographic and health survey; MICS: Multiple Indicator Cluster Survey; SPA: Service Provision Assessment; LiST: Lives Saved Tool.

## Competing interests

The authors declare that they have no competing interests.

## Author contributions

CH, PW, TM, MM, GM, AR, GT, JS, and SP conceived the quality management and the overall EQUIP approach. CH, PW, FM, SP, and the EQUIP Study team adapted the methodology and oversaw the implementation. CH, PW, AR, and SP drafted the manuscript. All authors read and approved the final manuscript.

## **EQUIP Study Team:**

Jennie Jaribu

Yovitha Sedekia

Pedro Arafumin

Silas Temu

Monica Okuga

Darious Kajjo

Rogers Mandu

Joseph Akuze

Hudson Balidawa

Ulrika Baker

Tara Tancred
