# Erratum to: Expanded Quality Management Using Information Power (EQUIP): protocol for a quasi-experimental study to improve maternal and newborn health in Tanzania and Uganda

**DOI:** 10.1186/s13012-015-0343-9

**Published:** 2015-10-29

**Authors:** Claudia Hanson, Peter Waiswa, Tanya Marchant, Michael Marx, Fatuma Manzi, Godfrey Mbaruku, Alex Rowe, Göran Tomson, Joanna Schellenberg, Stefan Peterson, the EQUIP Study Team

**Affiliations:** Department of Public Health Sciences, Karolinska Institutet, Stockholm, Sweden; Department of Disease Control, London School of Hygiene and Tropical Medicine, London, United Kingdom; Makerere University, College of Health Sciences, School of Public Health, Kampala, Uganda; Evaplan GmbH the University of Heidelberg, Heidelberg, Germany; Ifakara Health Institute, Dar-es-Salaam, Tanzania; Malaria Branch, Division of Parasitic Diseases and Malaria, Center for Global Health, Centers for Disease Control and Prevention, Atlanta, USA; Department of Learning, Informatics, Management, Ethics; Karolinska Institutet, Stockholm, Sweden; Department of Women’s and Children’s Health, International Maternal and Child Health Unit, Uppsala University, Uppsala, Sweden

Unfortunately, the original version of this article [1] contained mistakes. A portion of the text in the body of the article was supposed to comprise Table 2 (Table 1 here) and can be found below:

**Table 1:** Primary indicators

Primary coverage indicators
% of women delivering in a health facility (institutional delivery)
% of livebirths breastfed within one hour after delivery (immediate breastfeeding)
Primary quality indicator
% of livebirths where oxytocin was administered within one minute after delivery
Primary knowledge indicators
% of women knowing danger signs for pregnancy and newborn babies

5 additional files were also missing from the article. The details of these can be found as follows:

## Additional files

Additional file 1:
**Maps of the implementation and comparison areas in Uganda and Tanzania.** (DOCX 367 kb) Additional file 2:
**Implementation strategy.** (DOCX 275 kb) Additional file 3:
**Example of a run-chart.** (DOCX 221 kb) Additional file 4:
**Definitions.** (DOCX 20 kb) Additional file 5:
**Context documentation.** (DOCX 183 kb)
