# Supplementary material for: Erratum to: Expanded Quality Management Using Information Power (EQUIP): protocol for a quasi-experimental study to improve maternal and newborn health in Tanzania and Uganda
Source: Implement Sci. 2015 Oct 29;10:152. doi: 10.1186/s13012-015-0343-9 (PMC4627429; doi:10.1186/s13012-015-0343-9)
Supplement: Additional file 1: — Maps of the implementation and comparison areas in Uganda and Tanzania. (DOCX 367 kb) [file 13012_2015_343_MOESM1_ESM.docx]

**Manuscript Annex I** **Maps of the implementation and comparison areas in Uganda and Tanzania**

**Expanded Quality Management Using Information Power (EQUIP): protocol for a quasi-experimental study to improve maternal and newborn health in Tanzania and Uganda**

Hanson C, Waiswa P, Marchant T,  Marx M, Manzi F, Mbaruku G, Rowe AK, Tomson G, Schellenberg J, Peterson S, and the EQUIP Study Team.


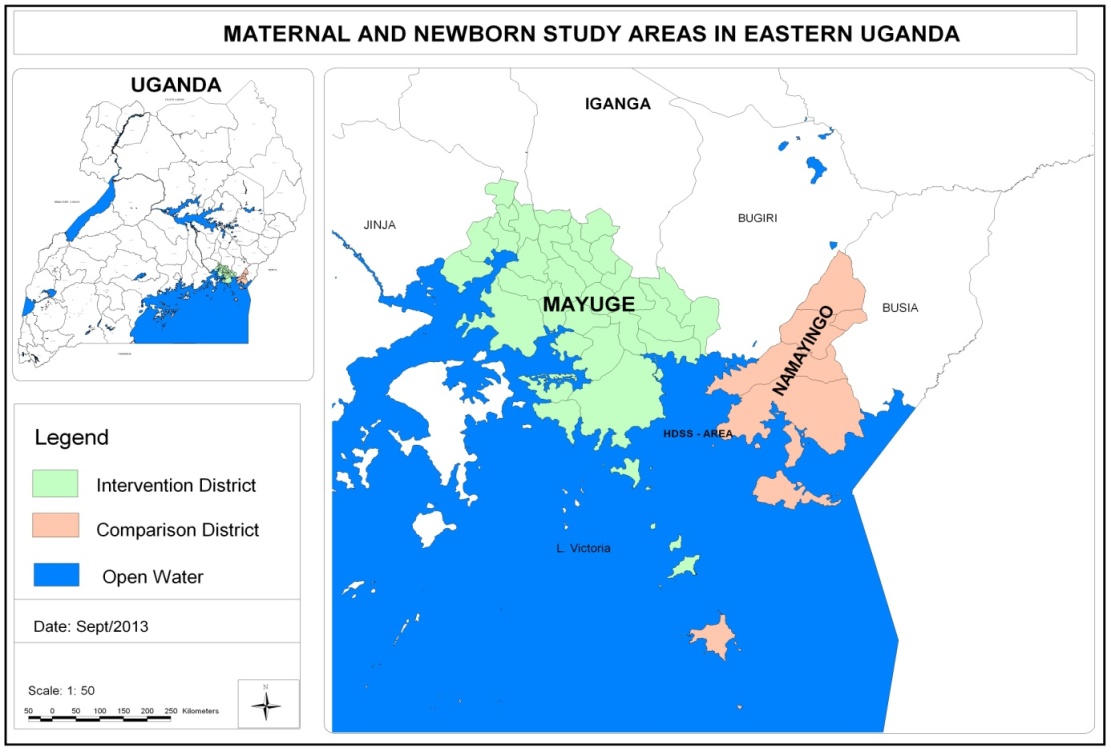


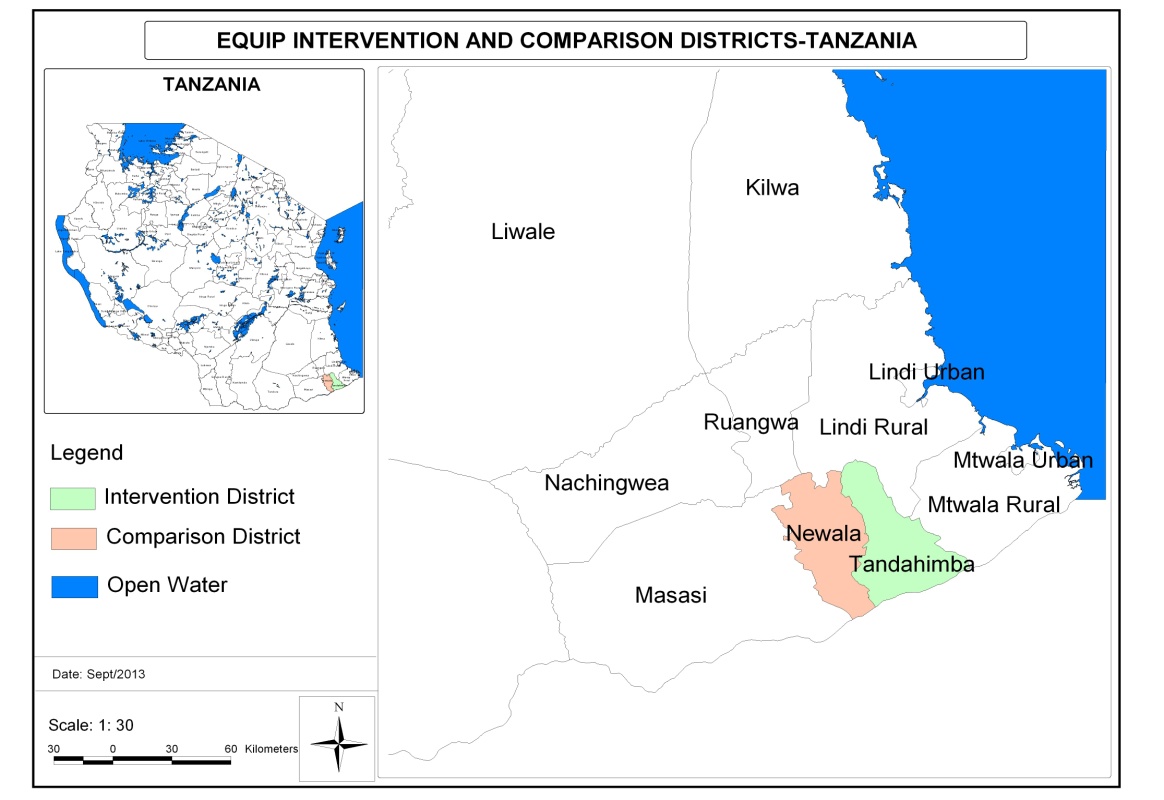
Figure 1: Implementation and comparison districts in Uganda

Figure 2: Implementation and comparison districts in Tanzania
