# Supplementary material for: Erratum to: Expanded Quality Management Using Information Power (EQUIP): protocol for a quasi-experimental study to improve maternal and newborn health in Tanzania and Uganda
Source: Implement Sci. 2015 Oct 29;10:152. doi: 10.1186/s13012-015-0343-9 (PMC4627429; doi:10.1186/s13012-015-0343-9)
Supplement: Additional file 2: — Implementation strategy. (DOCX 275 kb) [file 13012_2015_343_MOESM2_ESM.docx]

**Manuscript Annex II Implementation strategy**

**Expanded Quality Management Using Information Power (EQUIP): protocol for a quasi-experimental study to improve maternal and newborn health in Tanzania and Uganda**

Hanson C, Waiswa P, Marchant T,  Marx M, Manzi F, Mbaruku G, Rowe AK, Tomson G, Schellenberg J, Peterson S, and the EQUIP Study Team.

**Implementation strategy**

The implementation strategy in both Uganda and Tanzania is aligned to the government structures such as the District Health structure and Social Development Office’s structure. The main implementers are the District Health Teams (DHT) in Uganda and the Council Health Management Teams (CHMT) in Tanzania^[[1]](#footnote-1)^. In addition, in Tanzania the Social Development Department is included in the strategy to support the community component of EQUIP. Two EQUIP coordinators, one medical doctor and one social scientist, are supporting the strategy in each of the countries Tanzania and Uganda.

The quality management (QM) approach with its specific aims and objectives are outlined in the EQUIP quality improvement charter which was developed within the EQUIP consortium. The quality improvement charter sets the main indicators and improvement topics (see below). This charter guides the content of the learning sessions and mentoring throughout the districts for the whole implementation period.

**Training**

The EQUIP team members and key district support staff are trained by international trainers with extensive quality improvement experience during a four-day training. Other mentors at the sub-district level are trained by the EQUIP mentors during a two-day introduction training. Most of the training of mentors and the quality improvement teams (QITs) is learning-by-doing, as they are supported throughout implementation periods through mentoring and coaching visits. An external consultant also provides quality improvement mentorship and training to the teams in Tanzania and Uganda.

**Quality improvement teams and their composition**

Districts, health facilities and communities are encouraged to form QITs. The district QIT includes all members of the district health management teams in Uganda and Tanzania. In hospitals and large health facilities, QITs are comprised of 6–8 members, including staff from all critical departments (maternity, pharmacy, laboratory, in-charge of the facility and others).

At lower level health facilities, the QIT includes all health providers, typically 2–4 health providers. The composition of the community QITs is slightly different in Tanzania and Uganda.

In **Uganda**, QITs are established at the “parish” level. A parish typically includes 7–8 villages. Two volunteers, preferably members of the village health teams (VHT) are selected in village meetings together with village leaders so that 14–16 community volunteers from the villages form the parish QIT. In villages where no VHT is yet active, EQUIP, together with village leaders, holds sensitization meetings so that the village could select two volunteers. The selection criteria used for the volunteers was adopted from the Ministry of Health’s VHT selection criteria.

In **Tanzania**, as in Uganda, two volunteers are selected from the each of the 157 villages. Volunteers are selected on the basis of their education being preferably at a secondary school level, being a permanent village citizen, on the recommendation of the village officials, and through village meetings. A large number of volunteers are already working with other volunteer projects. The two volunteers from each village form a QIT. QITs from 15–18 villages form a cluster, which meet monthly to exchange with one another. Clusters are supervised by government extension workers who, by profession, oversee activities on behalf of the Departments of Education and of Community Development for local government authorities^[[2]](#footnote-2)^. Within EQUIP, they expand their activities to cover all wards in their cluster and supervise EQUIP volunteers on a volunteer basis. Three or four clusters then come together for learning sessions, making an improvement collaborative. A total of 10 clusters are carved out on the basis of health facility catchment areas. These are not in line with the Tanzanian administrative structures (wards and division), but in with a structure of support by extension workers which are part of the department of education and community development of the local government authorities^[[3]](#footnote-3)^.

The QITs at the district, health facility and community levels are encouraged by the mentors to meet at least monthly. Community-QITs (cluster and parish level) hold meetings together with one member of staff from the health facilities whose catchment falls within the cluster or parish.

**Support structure**

The support to the QITs in health facilities and communities is given through coaching and mentoring visits by EQUIP coordinators and involved district own staff.

Mentoring and coaching is done on a monthly basis. Teams are encouraged to engage in their own problem solving and to overcome barriers to maternal and newborn health by generating and testing change ideas. During the first year of implementation supervision has largely been done by the district support staff together with EQUIP members. Gradually, with improved confidence among the teams and mentors, the established cascade support system (see Figures 1 and 2) is increasingly used.

In **Uganda,** EQUIP staff includes the principal investigator, the EQUIP coordinator (medical professional) and assistant coordinator (social scientist). There tasks are the 1) development of the intervention and guidance, 2) monitoring, evaluation including implementation research, 3) mentoring and coaching of improvement teams at different levels.

The district own staff is involved in mentoring and coaching (two health facility mentors and two district community mentors). In addition four health sub-district mentors and 30 community mentors were trained to support the health facility and community teams in the sub-district.

At lower levels, a mentoring structure at sub-county level (four sub-district health facility mentors, and 30 sub-county community mentors) are also included to establish a cascade support structure. The village volunteers are member of the village health committees.

Off the 42 health facilities in the district, three were excluded because of reasons of accessibility and costs (the island facilities). A total of 39 facilities were initially included into the QI but nine dropped off during the first year as they provided either no delivery services or had inadequate staffing (less than 2 people) to form a QIT. Thus there were 30 active teams in Uganda, out of them 27 public facilities and three faith-based.

Figure 1: QI structure in Uganda

(QI: Quality Improvement, EQUIP: Expanded Quality management using Information Power)

In **Tanzania**, EQUIP staff (one medical professional and one social scientist under the guidance of the EQUIP main investigator) provide the primary support to both the health facility district mentor and the community district mentor (in dotted lines). In addition, the EQUIP staff mentor and support the QI work of the CHMT directly. The health facility district mentor, together with EQUIP staff, conducts mentoring and coaching meetings with the CHMT and each health facility monthly. The community district mentor directly oversees the ten extension workers who supervise each cluster. The EQUIP staff visit some of the QITs at cluster meetings to ensure that the extension workers are performing well and to help address any challenges that may arise.

All facilities operating in Tandahimba and providing reproductive health services were included in the QI work in Tanzania (32 off 34).

Figure 2: QI structure in Tanzania

(QI: Quality Improvement, EQUIP: Expanded Quality management using Information Power)

**Learning sessions and their content**

The work of the QITs is supported by quarterly one-day workshops called “learning sessions” (also see definitions in Annex IV). These are held separately for the district health teams, the health facilities, and the communities. Learning sessions at the health facility and community levels are organized by sub-district, where teams come together as a collaborative each with approximately 80-100 participants. The EQUIP coordinators organize, prepare and facilitate these sessions together with the district mentors and the extension workers.

Learning sessions include education on various topics related to maternal and newborn, but most importantly, they facilitate group discussions with the aim to develop improvement strategies. The initial learning sessions during 2012 also introduced principles of QM and the Plan-Do-Study-Act (PDSA) cycle, as well as the EQUIP project with its aims and objectives. At the health facility level, additional short trainings on topics such as post-partum care or neonatal resuscitation are prepared by the EQUIP coordinators and aim at complementing knowledge around specific improvement areas.

By providing teams with similar problems the opportunity to share experiences, the learning sessions facilitate an exchange between QITs to assist their generation of change ideas, encouraging and enabling the QITs to find their own solutions to problems related to the topic presented in their respective health facilities/villages. The use of PDSA cycles guides the creation of work plans, which highlight a clear objective related to the topic, an improvement strategy to be tested and indicators that will allow for testing of the strategy and monitoring of the strategy once it is applied at scale. Work plans are to be developed in learning sessions and formalized during follow-up mentoring and coaching meetings.

**Selection and roles and responsibilities of mentors**

In Uganda, mentors for both the health facility and community improvement work are chosen from the District Management Office using criteria such a having: (1) good rapport with the formal health sector; (2) experience working with the village health teams; (3) good clinical skills; and (4) an interest in improvement work.

In Tanzania, the selection of health facility mentors is done with the District Management Office, and the selection of the community-level mentor was done by the Community Development Office.

The main roles and responsibilities of the district level mentors in both countries are to facilitate meetings and learning sessions and to mentor and coach the QITs during the monthly mentoring and coaching visits. In addition, they oversee the work of the lower level support structure. Some of the mentoring and coaching visits are carried out together with the EQUIP quality improvement coordinators. District and sub-district level mentors are also included in the preparation and conduct of the learning sessions.

**Improvement Work**

**Selection of improvement topics – the Quality Improvement Charter**

The selection of improvement topics has been based on standard publications from WHO and partners such as the recent publication of essential maternal and newborn health interventions, commodities and guidelines [[1](#_ENREF_1)] and previous guidelines [[2](#_ENREF_2)] as well as country policy guidance of which interventions should be available in health facilities [[3-5](#_ENREF_3)].

Furthermore, implementation levels of key maternal and newborn health interventions is reviewed based on data from national household and facility surveys from both countries [[6-9](#_ENREF_6)]. In addition, in southern Tanzania, results from previous health facility and household surveys were used [[10](#_ENREF_10), [11](#_ENREF_11)]. During a consultative process within the technical maternal and newborn expert team of EQUIP, priority interventions were chosen based on two criteria: (1) interventions known to have a high impact on the burden of morbidity and mortality and (2) interventions where data suggested low or medium levels of implementation. We prepare ranking lists of priority interventions with roughly ten improvement topics for the five different levels of the health care system: (1) community; (2) lower level first-line facilities (health centre IIs in Uganda and dispensaries in Tanzania); (3) higher level first-line facilities (health centre IIIs and IVs in Uganda, health centres in Tanzania); (4) hospitals; and the (5) district management structure. These pre-prepared lists are used during learning sessions to introduce and guide the selection of priority topics for improvement. New topics are often selected by the EQUIP project members based on data of current performance generated from the continuous survey and guided by its potential impact on mortality. QITs can, however, also include other improvement topics based on local knowledge of low levels of implementation or major problems in their respective settings.

**Generation of change ideas**

During the learning sessions and supervision visits the teams are encouraged to use various quality improvement methods. These include brainstorming, fishbone analyses (see Figures 3), and other mapping techniques to highlight the complexity of the problems for which improvement strategies—change ideas—will be created to address. The use of PDSA cycles guides the creation of work plans, which highlight a clear objective related to the topic, a change idea to be tested and indicators that will allow for testing of the change idea and monitoring once the change idea is applied at scale.


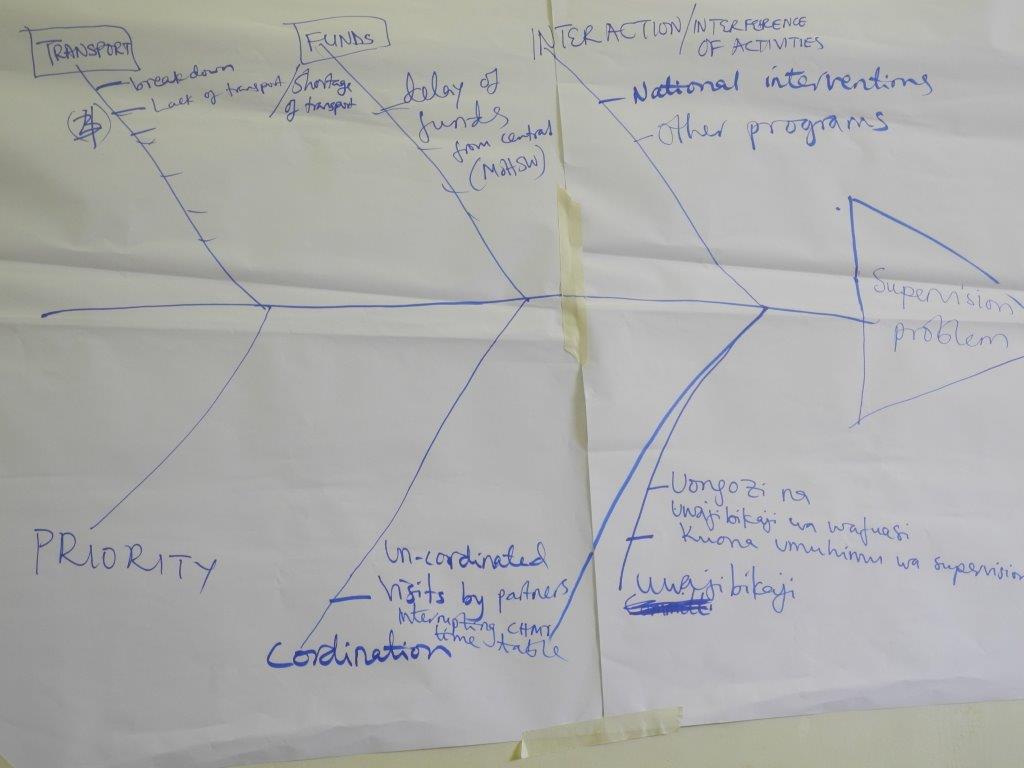


Figure 3: Problem analysis on district supervision to lower level facilities using fishbone diagram

**Indicators for monitoring at health facility and community levels**

Indicators for monitoring improvement strategies are prepared together with the list of improvement topics. A few were internationally-recognized standard indicators of health care coverage such as the proportion of mothers delivering with skilled attendance/institutional birth, four or more antenatal care visits, or uptake of postnatal care. Other indicators are more process oriented, such as the content of birth preparedness plans or adherence to the three components of Active Management of Third Stage of Labour. Wording of indicators was aligned with wording used in the household survey indicator list developed for the continuous health facility and household surveys of EQUIP, to facilitate a comparison between facility and survey data. Indicators based on the health facility records, use as a denominator “expected births in calendar year” rather than live births in the year prior the survey. If improvement topics are chosen by the teams where no indicator is available, the EQUIP coordinators and mentors assist them in identifying the best indicator to measure progress. The QITs also collect more detailed data to follow their specific improvement topic.

**Preparation, development and piloting of the EQUIP intervention**

The EQUIP approach was developed and piloted together with the district health teams (DHT and CHMT) during a one-year preparation phase (Nov 2010-Oct 2011). This period included a 4-day QI training for EQUIP project members and selected district staff led by international QM experts. The pre-testing phase was done from August 2011 to October 2012 to develop the overall structure and gain first experience. After this the approach was scaled up over a period of 1 year to all health facilities and communities in both countries.

Sensitization meetings at the district, sub-district and village levels are held to introduce the district authorities and the population to the aims of EQUIP. These meetings include information on the main objectives of the EQUIP project and the principles of quality improvement.

In both countries, health facilities and villages in one sub-district are chosen to start implementation first. The findings and experiences from these first implementation sites are then used to inform the further roll out. The initial implementation period also led to a further refinement and adaptation of the support structures. The quick improvements seen in coverage of certain interventions encourage the district teams to engage with EQUIP. For example in Uganda, measuring blood pressure during every antenatal care visit increased during one month from zero to 80% in the 14 health facilities included in the initial implementation area. Plotting progress in run-charts is seen a very powerful tool to raise commitment within health workers, district managers, and community members.

1. The DHT in Uganda and the CHMT in Tanzania are the official government structures put in place to support the district health system. These teams are responsible for the planning and implementation of all health related aspects in their districts. As both countries, Uganda and Tanzania, have been undergoing public sector reform for many years and have decentralised their health system (characterised as decentralisation by devolution), the teams are the main decision makers at the district level and answerable to the local government structure. [↑](#footnote-ref-1)
2. Extension workers are government employees within the Department of Education and Community Development. Their main mandate and duties within the government structure fitted well with the EQUIP goals of work at the community. An additional asset was they had received motorcycles for their other duties, which assist in their transport for EQUIP supervision [↑](#footnote-ref-2)
3. [↑](#footnote-ref-3)
