# Supplementary material for: Erratum to: Expanded Quality Management Using Information Power (EQUIP): protocol for a quasi-experimental study to improve maternal and newborn health in Tanzania and Uganda
Source: Implement Sci. 2015 Oct 29;10:152. doi: 10.1186/s13012-015-0343-9 (PMC4627429; doi:10.1186/s13012-015-0343-9)
Supplement: Additional file 3: — Example of a run-chart. (DOCX 221 kb) [file 13012_2015_343_MOESM3_ESM.docx]

**Manuscript Annex III Example of a run-chart**

**Expanded Quality Management Using Information Power (EQUIP): protocol for a quasi-experimental study to improve maternal and newborn health in Tanzania and Uganda**

Hanson C, Waiswa P, Marchant T,  Marx M, Manzi F, Mbaruku G, Rowe AK, Tomson G, Schellenberg J, Peterson S, and the EQUIP Study Team.

The following picture shows a run chart for the process indicator “blood pressure measuring during antenatal care” which was introduced within the early work of EQUIP in Uganda.

The flip chart paper includes the graph (run chart) showing the number of pregnant women coming for antenatal care whose blood pressure has been checked per months. The strategy (change idea) to improve implementation such as “acquiring a new blood pressure machine” and “continuous coaching” are presented in the dotted bubbles.


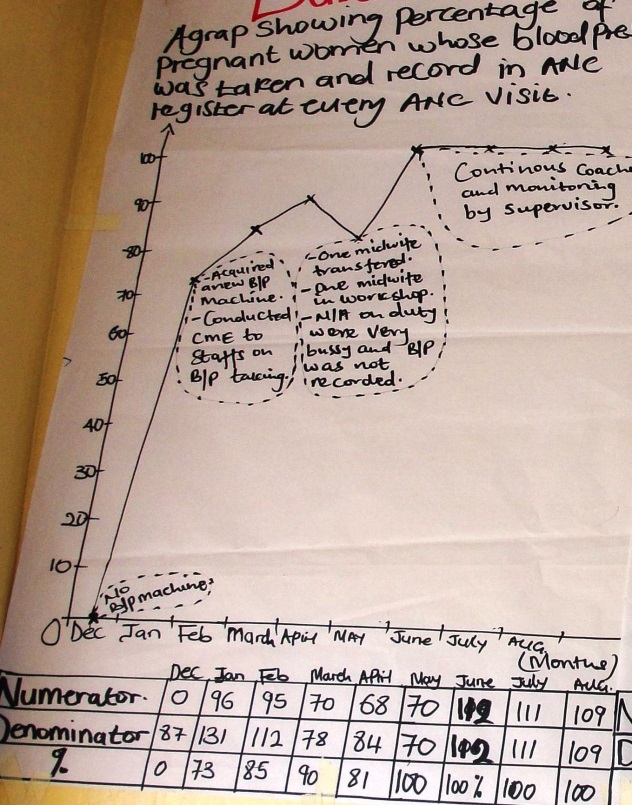


Figure1: Run chart on measuring blood pressure during antenatal care
