# Supplementary material for: Erratum to: Expanded Quality Management Using Information Power (EQUIP): protocol for a quasi-experimental study to improve maternal and newborn health in Tanzania and Uganda
Source: Implement Sci. 2015 Oct 29;10:152. doi: 10.1186/s13012-015-0343-9 (PMC4627429; doi:10.1186/s13012-015-0343-9)
Supplement: Additional file 4: — Definitions. (DOCX 20 kb) [file 13012_2015_343_MOESM4_ESM.docx]

**Manuscript Annex IV Definitions**

**Expanded Quality Management Using Information Power (EQUIP): protocol for a quasi-experimental study to improve maternal and newborn health in Tanzania and Uganda**

Hanson C, Waiswa P, Marchant T,  Marx M, Manzi F, Mbaruku G, Rowe AK, Tomson G, Schellenberg J, Peterson S, and the EQUIP Study Team.

Improvement collaborative A group of improvement teams working on a common improvement topic.

Action period The period where the quality improvement team implement their change ideas. This also includes continuous monitoring with agreed indicators and continuous assessment of results.

Change ideas Describes the ideas generated to solve a certain problem identified. Once a change idea has been tested and proven to be effective, it can be adopted by the quality improvement team responsible and applied at scale.

Coaching and mentoring Describes a process of support that aims to assist teams and individuals engaged in quality improvement activities to apply appropriate methodologies to create improvement. The main aspects are to listen, reflect, support, and advise.

Learning session Workshop that brings together representatives from quality improvement teams together to work on the same improvement topic (a collaborative). It is designed to allow participants to share experiences and to learn from each other and offers an opportunity to introduce new topics for improvement.

Improvement topic In our context evidence-based interventions that are known to generate improvement in the relevant area. The application of these proven interventions often needs local adaptation to be performed reliably, hence the need for change ideas

Quality improvement charter Outlines the plan for the improvement work with its overall aims and objective and indicators for monitoring. It also includes the way the improvement work will be delivered, the time frame, and the available resources.

.
