# Supplementary material for: Erratum to: Expanded Quality Management Using Information Power (EQUIP): protocol for a quasi-experimental study to improve maternal and newborn health in Tanzania and Uganda
Source: Implement Sci. 2015 Oct 29;10:152. doi: 10.1186/s13012-015-0343-9 (PMC4627429; doi:10.1186/s13012-015-0343-9)
Supplement: Additional file 5: — Context documentation. (DOCX 183 kb) [file 13012_2015_343_MOESM5_ESM.docx]

**Manuscript Annex Context documentation**

**Expanded Quality Management Using Information Power (EQUIP): protocol for a quasi-experimental study to improve maternal and newborn health in Tanzania and Uganda**

Hanson C, Waiswa P, Marchant T,  Marx M, Manzi F, Mbaruku G, Rowe AK, Tomson G, Schellenberg J, Peterson S, and the EQUIP Study Team.

**Introduction**

The comprehensive analytical approach and the plausibility design, which will be used for EQUIP demands measurement of indicators along the line from input to process to outcome and finally impact measures; complemented by contextual factors. The household and health facility surveys will primarily collect data on outputs and outcomes along the continuum of care. The impact of the intervention will be modeled using the LiST tool. Thus additional methods are needed to collect input indicators such as available funding for maternal and newborn health care and the information on strategies and policies.

Moreover the plausibility approach comparing the differences between intervention and comparison district demands a close follow up of contextual factors. The analysis of contextual factors is essential for the final judgment whether improvements in health indicators are likely to be due to the intervention or rather other factors. Victora et al. [[1](#_ENREF_1)] in their publication in the Lancet 2011 outlined factors affecting maternal and child health and nutrition. This publication can serve as a guide and framework for the follow up of context.


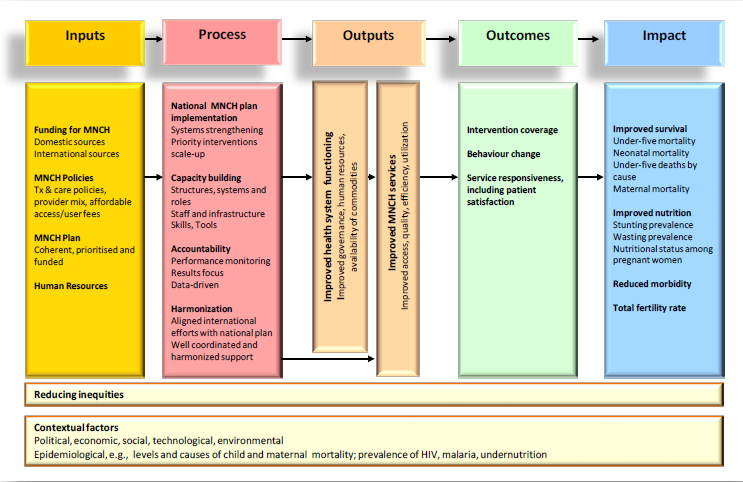


**Figure 5: Conceptual framework for evaluation**

See also: <http://www.jhsph.edu/bin/a/d/common_evaluation_framework.pdf>

Or: <http://www.internationalhealthpartnership.net//CMS_files/documents/monitoring_and_evaluation_of_health_systems_strengthening_-_an_operational_framework__EN.pdf>

**Figure 2: Outline of factors affecting maternal and child health and nutrition (Victora et al. Lancet 2011)**

The two frameworks presented suggest thus the follow of the following factors for context and input:

**Indicators of context**

- General socioeconomic and other contextual factors (such as poverty, employment, food production, fertility)
- Routine health services (such as health facilities, hospitals, human resources)
- Interventions in other sectors (such as water, sanitation, education, transportation)
- Other health programmes (such as family planning, food distribution, bednets)
- Coverage and impact of other health related intervention

**Indicators of input**

- Available funding for health and particular maternal and newborn health
- Policies
- Planning and strategising
- Human resources

The information to obtain for measuring input and context indicators might slightly overlap and also the way how the information will be obtained. The following list was prepared on the basis of the publication from Victora et al. [[1](#_ENREF_1)], the INSIST table of follow up of context, and the framework on evaluation from Bryce et al. [[2](#_ENREF_2)].

**Table: List of indicators / information for follow up of context and inputs**

| **Indicator /Information** | **Input /context** | **Possible source /Method to obtain information** |
| --- | --- | --- |
| **General socioeconomic & other contextual factors** | | |
| Poverty / wealth index | context | -Annual District reports including information on sales of cash crops  -Demographic and health surveys  -national reports such as UNDP development reports and others  --Inclusion of wealth index in HH-survey  -Census TZA 2012 |
| Education | context | -Annual Report of education team  -inclusion of education level in HH-survey |
| Population density  Fertility  Family size  Ethnic group | context | -Census / DHS data  -Inclusion of demographic data /fertility to HH- survey  -Inclusion of question concerning ethnic group to HH-survey  -inclusion of geographical positioning to HH-survey |
| Water supply  Sanitation | context | -annual district report from water /sanitation department |
| Transport | context | Reports / car and motor cycle registration  HH-survey availability of bicycle in household |
| Mobile phones | context | HH-survey availability of mobile phone in household |
| natural and political disasters such as droughts, floods | context | Reports, newspapers |
| **Baseline Health Characteristic** | | |
| Under-5-Mortality | context | INSIST /UNEST DHS/Census |
| Maternal and newborn mortality | context | INSIST/UNEST data |
| Prevalence of malnutrition | context | DHS |
| HIV prevalence | context | IN TZA: AIS 2010/11 and Uganda AIS 2011 but only regional, not district data available  Data from PMTCT as proxy |
| Malaria transmission | context | http://ipti.lshtm.ac.uk/questions/ (regional data available) |
| **Policy and governmental changes** | | |
| Health policy changes | Input /context | -Discussions with DHT/CHMT on major new policies, particular introduction of new intervention for maternal and newborn health |
| Governmental changes | Input /context | -Interview with DHT/CHMT maybe complemented by district administrative staff ( DED & DC in TZA) |

**Table: List of indicators / information for follow up of context and inputs, continued**

| **Health Service Characteristics** | | |
| --- | --- | --- |
| Number of hospitals, Health centres / dispensaries | Input/context | annual health plans and any annual health report |
| Health worker staffing patters | Input/context | Staffing assessed during HF-survey  (registers or pay-role registers often not reliable) |
| Drug supply | Input / context | Tracer drugs available assessed during HF-survey  Discussion / Interview with DHT/CHMT about availability of drugs |
| Infrastructure | Input / context | -HF survey  -rating from CHP in TZA |
| Information generation and utilisation | Input / context | -review of annual reports  -interview with DHT/CHMT |
| Baseline health care utilisation rates |  | DHS, HMIS |
| Availability of referral services | Input / context | HF-survey (ambulance, communication),  Discussion / Interview with DHT/CHMT about availability of ambulance and usage |
| **Presence of other projects and programmes that could affect health** | | |
| Malaria and HIV Programmes | Input / context | -Project reports  -Interviews with programme managers  -Training /support assessed during HF-survey |
| Micronutrients | Context | -Project reports  -Interviews with programme managers |
| Immunization | Context | EPI-documentation |
| Other maternal, newborn and child health programmes | Input / context | -Project reports  -Interviews with programme managers  -Training /support assessed during HF-survey |
| Community programmes (FP, malaria, maternal gender, ..) | Input / context | -Project reports  -Interviews with programme managers  -Training /support assessed during HF-survey |
| **Financing** |  |  |
| -Overall health budget  -specific budget for reproductive, maternal, newborn and child health | Input | -Health plans  -Interview with DHT/CHMT  -Interviews with project managers |
| **Governance and leadership** | | |
| Comprehensiveness of district planning | Input | Interview with DHT/CHMT on inclusive planning process involving key reproductive and child health specialists (such as RCH-CO, maternity staff)  & community groups / district health boards  & other specialists in district |
| Use of health data for planning | Input | Interview with DHT/CHMT on preparatory work for health planning |

Main methods to be used might be thus:

- Household survey (e.g. data on wealth quintiles, education)
- Health facility survey (e.g. data on staffing, drug availability, training received)
- Consultation of registers (such as pay role registers, car/motorcycle registration)
- Review of annual district reports (such as annual reports from department of education, water and sanitation, district health plans)
- Interview with DHT/CHMT (such as to investigate into drug availability/management, finances for health)
- Interviews with programme managers of other programmes (such as to know about their inputs in terms of funds, training, drugs,..)
- Previous research results (UNEST/INSIST)

The methodology includes qualitative and qualitative methods including survey results, exploitation of registers combined with interviews of key stakeholders and decision makers, programme manageress and others. The different sources and methods used will allow contrasting and validating qualitative and qualitative data and triangulation.

**Summary of methods and indicators**

**Inclusion of indicators in household survey**

- Wealth index (including possession of mobile phones and transport options)
- Education status
- GPS-data
- Number of children / fertility
- Ethnic group

**Inclusion of indicators in health facility survey**

- Staffing
- Training received by health facility staff
- Availability of key drugs
- Functioning fridge /other key equipment
- Community intervention in target area
- Training or other support for malaria, HIV, reproductive health, maternal and child health or other projects

**Annual district reports**

- General district reports
  - Economic report on cash crops sales, revenues, ..
- Report from education department
  - Number of primary and secondary schools, number of teachers
  - Ratio number of schools and teachers per population
- Report from department of water and sanitation
  - Have to look which indicators are available and useful
- Report from transport / roads department
  - Have to look which indicators are available and useful
- Annual health report
  - Crude immunization rates , utilization rates
  - Partner support (List of initiatives/NGOs/donors)
  - Financial information

**Interviews with DHT/CHMT**Main information to include in the questionnaire might be:

- Any important changes in the overall governance
- New policies, introduction of new interventions
- New donors/NGOs/partners and investment in health and particularly maternal and child health
- Changes in drug distribution system
- Any major initiatives in health facility infrastructure (new buildings, rehabilitation)
- Availability of transport / ambulances / functioning of referral system
- Training / capacity building in the field of reproductive health by health facility
- Training / capacity building in the field of quality management / quality improvement by facility
- Training / capacity building in the field community mobilization by facility
- Guided interview with open question in regard to planning process for the council/district plan,
  - who was involved (NGOs, community representatives, community boards/committees), district specialists in reproductive health, ...
  - which kind of data where used for guidance of priority setting and strategic decisions
  - how were conflicts between priorities resolved

**Interview with project staff from all projects/programmes/initiatives going on in the district**

Main information to be included in the questionnaire:

- What is the main focus and target group
- Where (geographically) is the project/programme/initiative implemented (by health facility)
- What are the main investments such as number of training, capacity building, infrastructure, drugs, human resources?

**Informal meetings with DHT/CHMT District coordination meetings**

Regular participation in district coordination meetings and frequent informal meetings with DHT/CHMT should complement the structured interviews. Documentation of any important new training, support, or any event in the field of reproductive health should complement the structured interviews which will be done once per quarter.

**References**

1. Victora, C.G., et al., *Measuring impact in the Millennium Development Goal era and beyond: a new approach to large-scale effectiveness evaluations.* The Lancet, 2011. **377**(9759): p. 85-95.

2. Bryce, J., et al. *Evaluating the scale-up for maternal and child survival: A common framework*. 2010 [cited 2011 June]; Available from: <http://www.jhsph.edu/bin/a/d/common_evaluation_framework.pdf>.
